# CMR Features in Cardiac Sarcoidosis

**DOI:** 10.1155/2011/702984

**Published:** 2011-07-13

**Authors:** Sparsh Prasher, Phong T. Lee, Marc Dweck, John R. Payne

**Affiliations:** ^1^School of Medicine, University of Glasgow, University Avenue, Glasgow G12 8QQ, UK; ^2^Department of Cardiology, Royal Infirmary of Edinburgh, 51 Little France Crescent, Edinburgh EH16 4SA, UK; ^3^Centre for Cardiovascular Science, The Queen's Medical Research Institute, University of Edinburgh, 47 Little France Crescent, Edinburgh EH16 4TJ, UK; ^4^Scottish Advanced Heart Failure Service, Golden Jubilee National Hospital, Beardmore Street, Clydebank, Glasgow G81 4HX, UK

## Abstract

Sarcoidosis is a multisystemic disorder of unknown aetiology characterised by the formation of noncaseating epithelioid cell granuloma involving various organ systems. Cardiac involvement has an important prognostic factor as it can present with life-threatening arrythmias and sudden death. Here, we present a case of cardiac sarcoidosis in a 46-year-old gentleman who presented with nonspecific signs and symptoms. We also discuss diagnostic difficulties especially when cardiac involvement is the only clinical sign. In this case, cardiac magnetic resonance (CMR) played an important role in the diagnosis and followup of our patient.

## 1. Introduction

Cardiac sarcoidosis is an important cause of death in patients with systemic sarcoidosis. Approximately 20–30% of patients with systemic sarcoidosis have evidence of granulomatous infiltration of the myocardium at autopsy [1]. The presence of scar tissue in the myocardium increases the risk of malignant arrhythmias, and up to 60% of fatalities from cardiac sarcoidosis present as sudden cardiac death [2].

## 2. Case Presentation

A previously well forty-six-year old Caucasian man presented with exertional dyspnoea and two cutaneous nodules on his left wrist which were biopsied and confirmed as sarcoid nodules. 

He was referred to the respiratory physicians for further investigations. Chest X-ray showed classical changes of pulmonary sarcoidosis, and serum ACE was mildly elevated at 92 U/L. He also described episodes of palpitation on exertion, ankle oedema, and progressively worsening breathlessness. However, the extent of his dyspnoea seemed disproportionate to that of his pulmonary involvement. An electrocardiogram (ECG) showed sinus rhythm with a prolonged PR interval (210 milliseconds), right bundle branch block, and left anterior fascicular block. 

Based on his clinical presentation and ECG abnormalities, heart failure secondary to cardiac sarcoidosis was suspected and he was referred to the cardiology department.

Twenty-four-hour ambulatory monitoring showed sinus rhythm though out with short runs of sinus tachycardia. Serum brain-natriuretic peptide (BNP) level was unremarkable at 21.3 pg/mL (normally less than 50 pg/mL). Percutaneous coronary angiography demonstrated normal coronary arteries, and endomyocardial biopsy showed “mild” and “nonspecific changes,” failing to confirm sarcoid infiltration. 

A CMR scan was subsequently performed to further examine the LV systolic function and assess for myocardial sarcoid infiltration. The left ventricle (LV) was nondilated and demonstrated moderate impairment of LV systolic function (ejection fraction 39%) associated with an akinetic but nonthinned interventricular septum. The right ventricular systolic function was also significantly impaired. Late gadolinium enhancement imaging showed several areas of myocardial hyperenhancement involving the anteroseptal subendocardium, subepicardium, and basal lateral epicardium.

The patient was initially treated with high-dose oral prednisolone therapy (40 mg/day) and referred to the tertiary heart failure centre. Following treatment, twelve lead ECG abnormalities improved with the only residual abnormality being right bundle branch block.

**Figure 1:**
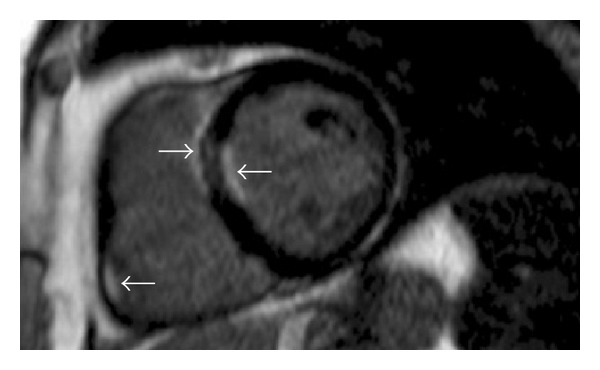
Gadolinium contrast-enhanced CMR scan; basal short-axis view demonstrating areas of hyperenhancement (arrows).

A second CMR scan was carried out 42 days after the initiation of his steroid therapy and showed marked improvement in right and left ventricular systolic function. Despite this, the areas of hyperenhancement seen in the first CMR scan remained. In addition, the RV surface of the inferior interventricular septum and RV free wall also appeared affected (Figure 1).

## 3. Discussion

Cardiac sarcoidosis is an important cause of death in patients with systemic sarcoidosis and should be considered in those who demonstrate cardiac symptoms and/or signs. Myocardial involvement is present in approximately 5 percent of patients with sarcoidosis although autopsy studies indicate subclinical cardiac involvement in 20 to 30 percent of cases [1]. Clinical manifestations of cardiac sarcoidosis depend on the location and extent of granulomatous infiltration and include conduction abnormalities, ventricular and supraventricular arrythmias, heart failure, valvular dysfunction, simulated infarction, and cor pulmonale.

Signs and symptoms of cardiac sarcoidosis are largely nonspecific, and, when cardiac involvement is the sole manifestation, diagnosis is often challenging. Previous attempts to identify sarcoid infiltration of the myocardium relied on transthoracic echocardiography, nuclear scintigraphy, and right ventricular endomyocardial biopsy [3]. The features of sarcoidosis on echocardiography include regional wall motion abnormalities which are nonspecific and often not manifest. Nuclear scintigraphy is also poorly sensitive and specific due to its suboptimal spatial resolution and artefacts whilst biopsy, when positive, is specific but provides unsatisfactory yield due to the patchy nature of infiltration [4].

CMR is a powerful noninvasive imaging modality which produces images with excellent spatial, soft tissue, and temporal resolution. Myocardial infiltration and scarring can be observed as late gadolinium hyperenhancement in the mid-wall and subepicardium. There seems to be a predilection for the lesions to affect the anterobasilar, anterolateral and lateral wall of the left ventricle. Furthermore, cardiac sarcoidosis is known to produce zones of thinning, segmental myocardial wall motion abnormalities, and subendocardial late gadolinium hyperenhancement similar to those seen in myocardial infarction but often in a noncoronary distribution [5–7]. CMR can be used to guide myocardial biopsy and improve its sensitivity and in certain cases may obliviate the need for biopsy completely.

In our patient, the first CMR scan demonstrated systolic dysfunction of both ventricles and both subepicardial and subendocardial late gadolinium hyperenhancement of the basal anteroseptal and basal anterolateral regions. This pattern appeared inconsistent with an ischaemic insult to the myocardium especially in the context of a normal coronary angiogram. Instead, supported by the normalisation of ventricular function following high-dose prednisolone, we felt it reflected myocardial infiltration and scarring secondary to sarcoidosis. 

This case highlights the importance of considering cardiac sarcoidosis in patients with systemic sarcoidosis when they present with symptoms such as heart failure. ECG may also be useful in indicating the possibility of myocardial infiltration. CMR offers a powerful noninvasive method of assessing myocardial fibrosis and infiltration, which can greatly assist in the diagnosis of sarcoid and in some cases eliminate the need for biopsy. In our patient, we demonstrated the role of CMR in diagnosing cardiac sarcoidosis especially when other investigations including endomyocardial biopsy failed to confirm sarcoid infiltration.

##  Consent

Written informed consent was obtained from the patient for publication of this case report and any accompanying images. A copy of the written consent is available for review by the Editor-in-Chief of this journal.

##  Conflict of Interests

The authors declare that they have no conflict of interests.

##  Authors' Contribution

J. R. Payne identified the patient, analyzed the images and edited the paper. S. Prasher acquired the images and ECGs, performed the literature search, and drafted the paper. M. Dweck and P. T. Lee contributed to literature search, writing and editing of the paper. All authors read and approved the final paper.
